# The efficacy of a new translational treatment for persecutory delusions: study protocol for a randomised controlled trial (The Feeling Safe Study)

**DOI:** 10.1186/s13063-016-1245-0

**Published:** 2016-03-11

**Authors:** Daniel Freeman, Felicity Waite, Richard Emsley, David Kingdon, Linda Davies, Ray Fitzpatrick, Graham Dunn

**Affiliations:** Department of Psychiatry, University of Oxford, Warneford Hospital, Oxford, OX3 7JX UK; Centre for Biostatistics, Institute of Population Health, Manchester University, Manchester Academic Health Centre, Manchester, UK; Academic Department of Psychiatry, Faculty of Medicine, University of Southampton, Southampton, UK; Centre for Health Economics, Institute of Population Health, Manchester University, Manchester Academic Health Centre, Manchester, UK; Nuffield Department of Population Health, University of Oxford, Oxford, UK

**Keywords:** Paranoia, persecutory delusions, schizophrenia, psychosis, cognitive therapy

## Abstract

**Background:**

Persecutory delusions (strong unfounded fears that others intend harm to the person) occur in more than 70 % of the patients diagnosed with schizophrenia. This major psychotic experience is a key clinical target, for which substantial improvement in treatment is needed. Our aim is to use advances in theoretical understanding to develop a much more efficacious treatment that leads to recovery in at least 50 % of people with persistent persecutory delusions. Our cognitive conceptualisation is that persecutory delusions are threat beliefs, developed in the context of genetic and environmental risk, maintained by a number of psychological processes including excessive worry, low self-confidence, intolerance of anxious affect and other internal anomalous experiences, reasoning biases, and safety-seeking strategies. The clinical implication is that safety has to be relearned, by entering the feared situations after reduction of the influence of the maintenance factors. We have been individually evaluating modules targeting causal factors. These will now be tested together as a full treatment, called The Feeling Safe Programme. The treatment is modular, personalised, and includes patient preference. We will test whether the new treatment leads to greater recovery in persistent persecutory delusions, psychological well-being, and activity levels compared to befriending (that is, controlling for therapist attention).

**Methods/design:**

The Feeling Safe Study is a parallel group randomised controlled trial for 150 patients who have persecutory delusions despite previous treatment in mental health services. Patients will be randomised (1:1 ratio) to The Feeling Safe Programme or befriending (both provided in 20 sessions over 6 months). Standard care will continue as usual. Online randomisation will use a permuted blocks algorithm, with randomly varying block size, stratified by therapist. Assessments, by a rater blind to allocation, will be conducted at 0, 6 (post treatment), and 12 months. The primary outcome is the level of delusional conviction at 6 months. Secondary outcomes include levels of psychological well-being, suicidal ideation, and activity. All main analyses will be intention-to-treat. The trial is funded by the NHS National Institute for Health Research.

**Discussion:**

The Feeling Safe study will provide a Phase II evaluation of a new targeted translational psychological treatment for persecutory delusions.

**Trial registration:**

Current Controlled Trials ISRCTN18705064 (registered 11 November 2015).

## Background

Persecutory delusions, a central problem in schizophrenia, are unfounded beliefs that others are trying to harm the person [1]. Nearly half of patients with persecutory delusions have major depression [2]. Persecutory delusions predict serious violence [3], suicide [4], and hospital admission [5]. It is well-recognised that treatments for persecutory delusions need significant improvement. The first line treatment, medication has effect sizes (standardised mean differences) varying between 0.33 and 0.88 (median = 0.44) [6], with problems of major side effects, poor compliance, and residual symptoms. In a review, Kennedy et al. [7] found that ‘almost 60 % of patients failed to achieve response after 23 weeks on antipsychotic drug therapy’. Meta-analysis for first generation psychological treatment (when added to medication) indicates an effect size of only 0.36 for delusions [8]. Psychological treatment is a valued treatment choice for patients, but problems of availability exist. For instance, in the United Kingdom only about 5 to 10 % of patients receive cognitive behavioural therapy (CBT) for psychosis [9]. Using advances in the understanding of the causes of persecutory delusions, our team have been developing a new targeted modular psychological treatment - called ‘The Feeling Safe Programme’ - with the aim of improving efficacy and deliverability.

### The translational studies leading to the trial

**Fig. 1:**
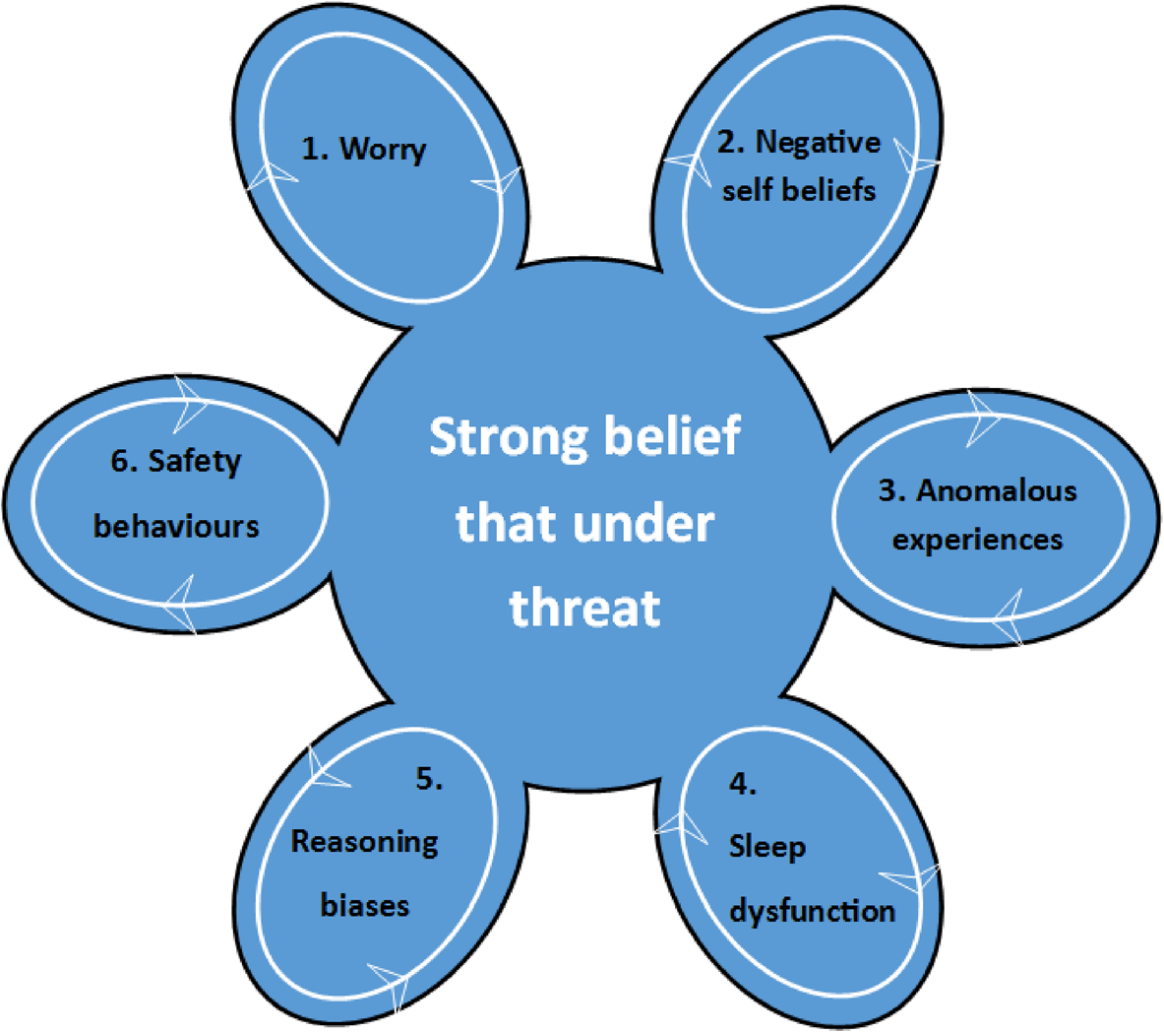
The maintenance of persecutory delusions [12]

At the core of a persecutory delusion is the belief that the person is unsafe [10]. New research shows that the heritability of paranoid thoughts is 50 % [11], indicating genetic and environmental risk leading to such fears. Once developed, the beliefs concerning danger are maintained by six key factors [12] (see Fig. 1). For example, worry brings implausible ideas to mind, keeps them there, and exacerbates the distress; negative self-beliefs lead the person to feel inferior and vulnerable; subjectively anomalous internal states (for example, dissociation, unexplained anxious arousal, and perceptual disturbances) provoke fearful explanations; disrupted sleep increases negative affect, mood dysregulation, and anomalous internal states; reasoning biases prevent the processing of alternative explanations; and safety-seeking (defensive) behaviours such as avoidance prevent the person receiving and processing disconfirmatory evidence that he or she is safe. Therefore treatment needs to target the maintenance factors before helping the patient to go into everyday situations and relearn that they are safe.

Our team have been developing and evaluating brief treatments targeting these maintenance factors. Brief, manualised interventions have been used in order to aid the theoretical interpretation, later dissemination, and the building of a combined treatment. The strongest test has been for reducing worry. A randomised controlled trial (‘The Worry Intervention Trial’) with 150 patients with persistent persecutory delusions was completed [13]. This had blind ratings and a 95 % follow-up rate. Targeting worry, in just six sessions, significantly reduced both worry and the persecutory delusions (both effect sizes = 0.5). A mediation analysis showed that two-thirds of the reductions in the delusions were due to reductions in worry. There were also significant increases in psychological well-being and reductions in overall psychiatric symptoms. A pilot randomised controlled trial (‘The Self-Confidence Study’) with 30 patients with persistent persecutory delusions, principally used techniques to enhance positive self-beliefs in order to limit the effects of negative self-beliefs [14]. Ratings were blind, and 100 % of the patients were followed up. Post treatment improvements were observed in positive self-beliefs (effect size = 1.0) and psychological well-being (effect size = 1.2) and reductions in negative self-beliefs (effect size = 0.24) and the delusions (effect size = 0.6). An assessor-blind, pilot randomised controlled trial (‘The Better Sleep Trial’) with 50 patients with persistent delusions and hallucinations showed that sleep can be substantially improved (effect size = 1.9) and that consequential benefits may exist in the levels of paranoia (effect size = 0.2) and quality of life (effect size = 0.5) [15]. A trial with several thousand university students with insomnia is now underway that will have sufficient power to test definitively the relation between sleep improvement and paranoia [16]. Two recent randomised controlled studies have shown the benefits of reducing reasoning biases in patients with delusions [17, 18]. For example, in a pilot clinical study with 31 patients with persistent delusions, the ‘Thinking Well’ reasoning intervention led to a reduction in delusional conviction (effect size = 0.6) compared to standard care [18]. Most recently, we have shown that going into feared situations (that is, reducing avoidance) while dropping safety-seeking behaviours that prevent the full processing of disconfirmatory evidence reduces delusions to a much greater extent than exposure alone (effect size = 1.3) [19]. All these elements have now been combined as part of a full intervention, called *The Feeling Safe Programme*, delivered in 20 sessions over 6 months. The feasibility of this treatment has been recently established in a case series, and indications exist of substantial clinical benefits for the patients [20]. The Feeling Safe Programme has been developed further on the basis of this case series.

### The new clinical trial

The primary aim now is to test the efficacy in a single centre of this new theoretically-driven treatment for persecutory delusions. The target group is those at most need: patients whose delusions have not responded to current treatment. The Feeling Safe Programme is anticipated to lead to 50 % of patients having recovery in persistent persecutory delusions. We will test the intervention against an equal time receiving befriending (called ‘Feeling Safe and Supported’) from the same therapists. Befriending has benefits for patients with psychosis and in the short-term is comparable to first-generation cognitive-behavioural psychological therapies for psychosis [21, 22]. This choice of comparison allows us to determine whether the Feeling Safe Programme has benefits over and above the extra time spent with a therapist, which is important to determine for future training needs and service provision.

The primary outcome will be conviction in the persecutory delusion, testing rates of recovery in the delusions (defined as conviction falling below 50 %, that is, greater doubt than certainty in the delusion) and dimensional reductions in conviction levels. The Feeling Safe Programme is hypothesized to lead to lower levels of conviction in the persecutory delusions compared to befriending. Key secondary hypotheses are that the Feeling Safe Programme, compared to befriending, will lead to improved psychological well-being and activity levels compared to befriending. The Feeling Safe Programme, compared to befriending, is also predicted to lead to lower levels of overall paranoia, total delusion severity, and suicidal ideation. The primary endpoint will be the 6-month outcomes (that is, post-treatment) but the persistence of effects at a longer follow-up will also be tested (12 months).

An explanatory component to the trial will be included. We will test whether changes in key maintenance factors (worry, self-beliefs, anomalous experiences, sleep, reasoning, safety-seeking behaviours) mediate change in delusions. We will also test whether working memory, illicit drug use, and levels of anger moderate treatment effects. We will record all service use, and other relevant health economic data, in order to carry out a health economic analysis.

## Methods

The trial has received ethical approval from an NHS Research Ethics Committee (South Central – Oxford B Research Ethics Committee; ref 15/SC/0508) and has been registered (Current Controlled Trials ISRCTN18705064). Informed consent will be obtained from all participants. A Data Monitoring and Ethics Committee (DMEC), Trial Steering Committee (TSC), and Patient Advisory Group (PAG) have been formed.

### Design

**Fig. 2:**
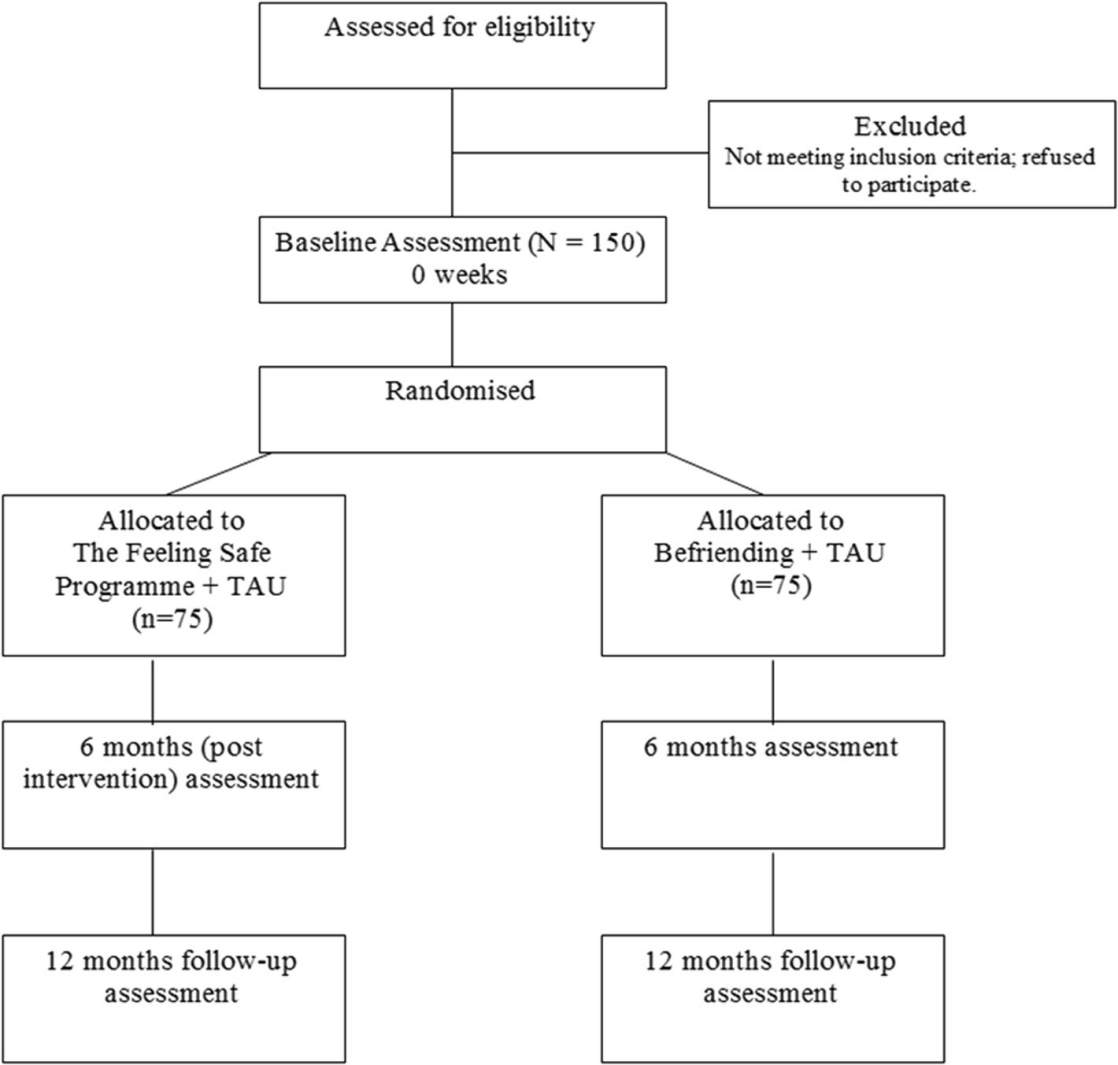
Trial flow diagram

The design is a parallel group randomised controlled trial with single blind assessment to test whether the new psychological treatment will reduce persecutory delusions more effectively than befriending (an attention control condition) (see Fig. 2). Standard care will be measured but remain as usual in both groups. Assessments will be carried out at 0, 6 (post treatment), and 12 months.

### Participants

Participants will be sought who have persistent persecutory delusions in the context of non-affective psychosis. The inclusion criteria are male or female, aged 16 years or above; persistent (at least 3 months) persecutory delusion (as defined by Freeman and Garety [1]), held with at least 60 % conviction; and primary diagnosis of schizophrenia-spectrum psychosis (non-affective psychosis). The exclusion criteria are current receipt of another psychological therapy; insufficient comprehension of English; primary diagnosis of alcohol, drug, or personality disorder; in forensic settings; organic syndrome; or learning disability. Referrals will be sought from Oxford Health NHS Foundation Trust and neighbouring NHS Trusts (for example, Northamptonshire Healthcare NHS Foundation Trust and Berkshire Healthcare NHS Foundation Trust).

### Randomisation and blinding

The trial assessor will be blind to group allocation, but the patients and trial therapists will not be. Patient consent and assessments will be carried out by the trial assessor. Randomisation will occur after completion of the baseline assessment. An online randomisation system has been written by the University of Oxford Primary Care Clinical Trials Unit. Randomisation using a permuted blocks algorithm, with randomly varying block size, will be stratified by therapist. Therapists will provide both interventions in order to reduce the confounding of therapist effects and increase statistical power. The trial co-ordinator will use the online system, after being provided by the trial assessor with basic patient details (date of birth, gender).

The trial coordinator will inform trial therapists who will then inform patients of the randomisation outcome, so that the research assessors remain blind to group allocation. Precautionary strategies to prevent breaks of the blind include the following: the patients being reminded by team members not to talk about treatment allocation; the assessor not looking at the patient’s clinical notes after the baseline assessment; and if an allocation is revealed between assessment sessions, then re-blinding with another assessor. We envisage concealment of treatment allocation from the trial assessor will be easier than treatment-as-usual comparison trials because all patients are receiving a psychological intervention from the same therapists.

### Assessments

Basic demographic and clinical data will be collected (for example, age, gender, ethnicity, and clinical diagnosis). The primary outcome measure will be conviction in the persecutory delusion (using a 0 to 100 % scale), assessed within the Psychotic Symptoms Rating Scale-Delusions scale [23]. Recovery is defined as the conviction in the delusional belief falling below 50 %; that is, there is greater doubt than belief in the delusion. Conviction greater than 50 % is a standard definition of the presence of a delusion (for example, [24]), although such beliefs are typically held with much greater certainty. For example, in our Feeling Safe Programme pilot study (n = 12), the initial conviction levels in the delusions showed a mean of 90 % (SD = 17) [20], and in a previous study with 100 patients with delusions, the mean conviction rating was 82 % (SD = 20) [25]. In the Worry Intervention Trial, at baseline, half of the 150 patients had 100 % conviction in the persecutory delusions [13].

Psychological well-being will be assessed by the Warwick-Edinburgh Mental Well-being Scale [26], health status by the EQ-5D-5 L (see http://www.euroqol.org/), quality of life by the Long Term Conditions Questionnaire (LTCQ) [27], and patient satisfaction using an adapted version of the CHOICE, a service user-led outcome measure [28]. Activity levels will be assessed using a step count and a time-budget measure [29]. We will also include measures of overall paranoia (Green et al. Paranoid Thoughts Scale) [30], suicidal ideation (Columbia-Suicide Severity Rating Scale) [31], and depression (Beck Depression Inventory) [32].

We will include the following as moderators: working memory [33], illicit drug use [34], and anger (Dimensions of Anger Reactions (DAR-5)) [35]. For mediation, we will include the following: the Penn State Worry Questionnaire [36], Brief Core Schema Scales [37], Specific Psychotic Experiences Questionnaire - hallucinations subscale (SPEQ) [38], Insomnia Severity Index [39], jumping to conclusions [40] and belief flexibility [18], and the Safety Behaviours Questionnaire – Persecutory Beliefs [41]. We will record service use and other relevant health economic data using an adapted version of the Economic Patient Questionnaire [42] (EPQ) that includes questions from the Client Service Receipt Inventory [43].

In collaboration with the McPin Foundation, qualitative interviews will be carried out with a small number of patients and family members about the Feeling Safe Programme to assess the acceptability of the experimental intervention.

### Adverse events

We will check medical notes at the end of a patient’s participation for serious adverse events, including but not limited to: 1. All deaths. 2. Suicide attempts. 3. Violent incidents (needing police involvement) and 4. Formal complaints about therapy. We will also record any such event that we become aware of during a patient’s participation. All hospital admission data will also be recorded. The DMEC will determine relatedness of an event to the trial based on a temporalrelationship and whether the event is unexpected or unexplained given the participant’s clinical course, previous history, and concomitant treatments.

### Psychological interventions

Both treatments are provided to patients individually in approximately 20 sessions over 6 months. Treatments will be provided by the trial clinical psychologists, with weekly supervision. The number of sessions and length will be recorded, sessions will be taped when patients are agreeable, and tapes will be rated for fidelity and competence. Patient beliefs about the potential effectiveness of the intervention that he or she receives will be assessed after the first session with the Credibility/Expectancy Questionnaire [44], and therapeutic empathy will also be assessed with a patient questionnaire [45].

In The Feeling Safe Programme, following an assessment, the patient is offered a menu of appropriate treatment modules. Typically three to four modules are completed, based on patient preference. The range of modules that can be offered are improving sleep, reducing worry, increasing self-confidence, reducing the impact of voices, improving reasoning processes, and behavioural tests for reducing fear beliefs. Befriending, called in the trial ‘Feeling Safe and Supported’ will follow a protocol devised by one of the trial team members (DK) that has previously been used in two large clinical trials for patients with psychosis over 20 sessions [21, 22]. Essentially, the aim is to simulate how a good friend would respond and involves a general focus on non-threatening topics (although patients are not actively dissuaded from talking about concerns), non-confrontation, empathy, and supportiveness.

### Statistical and economic analysis plan

A full statistical analysis plan will be written by the trial statisticians (RE, GD) prior to any analysis being undertaken. We will report data in line with the Consolidated Standards of Reporting Trials (CONSORT) 2010 Statement (http://www.consort-statement.org/consort-2010), showing attrition rates and loss to follow-up. All analyses will be carried out using the intention-to-treat principle with data from all participants in the analysis, including those who do not complete therapy. Every effort will be made to follow up all participants in both arms for research assessments.

Analysis will be conducted in Stata version 14 [46]. Descriptive statistics within each randomised group will be presented for baseline values. These will include counts and percentages for binary and categorical variables and means and standard deviations, or medians with lower and upper quartiles, for continuous variables, along with minimum and maximum values and counts of missing values. There will be no tests of statistical significance or confidence intervals for differences between randomised groups on any baseline variable.

Descriptive statistics will be used to summarize assessments of feasibility and acceptability in terms of recruitment, drop-out, and completeness of therapy.

The primary hypothesis for change in the primary outcome measure, conviction in the persecutory delusion (using a 0 to 100 % scale) at 6 months, will be analysed using a linear regression model allowing for the baseline measurement of outcome, severity of delusion, therapist and treatment assignment as fixed effects. To compare rates of recovery (scores falling below 50 %), we will use logistic regression models instead of linear models. Secondary outcome measures will be analysed using the same modelling approach. This includes analysis of the primary outcome and secondary outcomes at 12 months.

The mediation analysis will investigate putative mediational factors using modern causal inference methods [47, 48]. This involves using parametric regression models to test for mediation of the Feeling Safe Intervention on outcome through the putative mediators. Analyses will adjust for baseline measures of the mediator, outcomes, and possible measured confounders. We will include repeated measurement of mediators and outcomes to account for classical measurement error and baseline confounding, and where feasible, use instrumental variable methods (baseline covariate by randomization interactions as potential instruments) to investigate the sensitivity of the estimates to these problems and that of unmeasured confounding.

Moderators will be assessed separately by repeating the primary analysis models and including interaction terms between the randomised intervention and each moderator. The coefficient of the interaction term is a measure of whether the treatment effect differs between levels of the moderator.

Missing data on individual measures will be pro-rated if more than 90 % of the items are completed; otherwise the measure will be considered as missing. We will check for differential predictors of missing outcomes by comparing responders to non-responders on key baseline variables. Any significant predictors will be included in the analysis models. This accounts for missing outcome data under a missing at random assumption, conditional on the covariates included in the model. As a sensitivity analysis, we will assess whether treatment adherence is associated with missing data, and if it is associated, use inverse probability weights or multiple imputation to compare results.

An economic evaluation will estimate the cost per quality adjusted life year (QALY) gained from a health and social care perspective over the 1-year timeframe of the trial. An economic model will be used to explore the potential cost effectiveness of the intervention over the patient’s lifetime. A detailed analysis plan for the economic evaluation will be prepared by the trial health economist (LMD) prior to the analysis. This will be informed by exploratory analyses of the pooled baseline data and published literature.

For a recovery rate in delusions of 50 % in the Feeling Safe Programme, compared to 20 % with befriending, a study will have over 90 % power with 60 patients in each arm. The trial will, however, gain greater power by also examining change in delusion dimensional scores. If the standardised effect of the new intervention compared to befriending were smaller than 10 percentage points on the conviction scale (0 to 100 %) (d = 0.5), then we would not consider further development of the intervention to be worth pursuing. If the true effect size were this ten-point difference (SD = 20), then a two-sample t-test with a two-sided significance level of 0.05 would have 80 % power to detect a statistically significant effect with outcome data available for 64 participants per randomised arm. We aim to recruit 75 per arm. This conservatively allows for a drop-out of 15 %. Allowing for stratum membership and baseline levels of the measures in a more refined analysis of covariance will increase both statistical power and precision.

## Discussion

Over the past 15 years, a significant advance has occurred in the understanding of the causes of paranoia. This research has predominately been from a cognitive perspective [12,49–52]. The advance in knowledge is beginning to be translated into treatment. From our theoretical model, the clinical goal becomes to enable the patient to form a strong belief concerning current safety, thereby allowing the persecutory threat belief to dissipate. Hence the influence of the maintenance factors needs to be reduced and patients re-enter the threatening situations in order to learn directly that nothing adverse occurs. Toleration of the high anxiety, associated physiological arousal, and other anomalous experiences needs to occur. This learning of safety should allow a fundamental shift of attention away from activation of the negative valence system. From this perspective, clinical trials need to recruit patients on the presence of having persecutory delusions, and such delusions and related behaviours should become the main outcome. The Feeling Safe Study will be such an example. Translating our cognitive model, a series of studies of persistent persecutory delusions have shown the benefits of targeting the maintenance factors individually. The current trial aims to determine the efficacy, above that of simple therapist effects, of a full treatment based on the theoretical understanding. The potential is a substantial improvement in treatment for persistent persecutory delusions. Outcome results are expected in 2020.

## Trial status

The trial is due to start patient recruitment in February 2016.

## Abbreviations

CBT: cognitive behavioural therapy
CHOICE: Choice of Outcome In CBT for Psychoses
CONSORT: Consolidated Standards of Reporting Trials
CSRI: Client Service Receipt Inventory
DAR-5: Dimensions of Anger Reactions
DMEC: Data Monitoring and Ethics Committee
EPQ: Economic Patient Questionnaire (EPQ)
EQ-5D-3 L: EuroQual Five Dimensions Three Levels
LTCQ: Long Term Conditions Questionnaire
NHS: National Health Service
PAG: Patient Advisory Group
QALY: quality-adjusted life year
SPEQ: Specific Psychotic Experiences Questionnaire
TSC: Trial Steering Committee.
